# Some Human Lessons in War-Time

**Published:** 1916-10-14

**Authors:** 


### LIFE AND DEATH.

### Some Human Lessons in War-Time.

### IV. RICHES AND WEALTH.*

### THEIR LIMITATIONS, USE, AND IMPOTENCE.

Dr. Johnson declared that "money can neither open new avenues to pleasure nor block up the passages of anguish." The avenues to pleasure which are opened by the possession and use of money may, and often do, soon become a weariness of the flesh. Whenever money is used in the sense Dr. Johnson clearly meant, its fruits become like the apples of Sodom and Gomorrah, hateful and repellent. Similarly, money, no matter what its amount, cannot compensate the victim of irreparable wrong. Money cannot restore purity to the outraged; it cannot give back the life which has been compassed through an assassin. No amount of money can remove or efface injustice wantonly and cruelly inflicted upon the innocent. Considerations of this kind, and the experience of succeeding generations throughout the world's history, have led men of all types to agree that money is the root of most evil. Further than this, it may be said, as members of the medical profession often have evidenced in the course of their practice, that the possession of money in excess may tend indirectly to undermine health, to destroy character, to produce many forms of illness, including the loss of reason, and the destruction of life, frequently in the young, and not seldom in the aged, who have but a slight, if any, realisation of their spirituality. Such often become bankrupt in health owing to the habits of parsimony, of dissipation, of excessive craving after a continuous increase in their already large possessions; or from fear of the loss of their money from one of many causes; or from a host of strange and sometimes extraordinary illusions which not infrequently possess the over-rich, to whom money has become the beginning and end of existence.

### The Millionaire's Dissatisfaction.

We should say, as a matter of experience, that the inordinately rich have greater temptations and fewer chances of enjoying robust health and a quiet mind than probably any other class of men. At the best, when the novelty of the possession of money to excess has worn off, and experience has taught the limitations of the uses of money, a spirit of restless dissatisfaction frequently and increasingly possesses the millionaire, because, may be, he has expended much energy and continuous effort in the endeavour to make money fruitful in its results to himself. Those who value health, whom wisdom has taught the difference between living and existing, that is, in fact, the best types of the race, have no wish to be wealthy; and not a few of the best men in each generation have deliberately refused to accept the wealth opportunity unexpectedly opened out to them. A striking proof of the wisdom of this policy of deliberate rejection is the Lancashire proverb, "Clogs, boots, clogs."

Of course the young, and the relatively poor, and those without adequate means to enable them to live in comfort, not unnaturally would oppose as ridiculous or unfounded the views here set down. These statements are not mere opinions, but the actual teachings of the experience of men who have been blessed or cursed by the possession and responsibility of enormous fortunes. Who, capable of thought and understanding, can envy the positions of the wealthiest men of the day, in the conditions with which war has surrounded them and their possessions? The truth is that when a man or woman who is possessed of the knowledge of spirituality has sufficient means to pay for the actual cost of living in the circumstances of their class, they will rest content, feeling themselves free from a multitude of anxieties, temptations, and troubles which invariably attach to the possession of money in great quantities. It is the contented mind which is a continual feast. The possession of vast sums of money often proves a continual and increasing burden to its owner.

### The Conclusion of the Wise.

There is, however, another side to wealth, and that is its best side. There are wealthy persons of both sexes who have set themselves, from the highest motives, to employ their wealth to benefit their fellows, in the hope of leaving the world a little better than they found it. They have spent long days and laborious years in the attempt to solve the problem of how to effect this praiseworthy result, free from mistakes and without suffering the chagrin of finding their noblest conceptions, when reduced to practice, not only a disappointment but a failure. The most striking exemplification of the failure of the best-laid plans of this type is often seen in the results which follow large bequests of money for special purposes. Nowadays Parliament and the Law have done much to minimise failures of this kind, but examples occur still which enforce the conclusion of the wise, that the man with money should nerve himself to the task of giving his money away in his lifetime to approved objects, and to follow his gift with continuous interest so long as he lives. It is a strange but true testimony that money, when possessed to excess, seems to cling to the possessor, who often cannot nerve himself to part with any considerable sum, though the gift of it would only mean to him or her a book entry.

We have been considering wealth in the practical and every-day aspects in which it presented itself before the outbreak of war. The war has brought to the wealthy, as it has brought to many others, an

* Previous articles appeared on September 23 and 30, and October 7.

education in world truths. The enormous expenditure entailed by the great war has impressed upon the whole community, and especially upon the man of business who is also a man of wealth, the value of money. The pleasure of possession has come to him with telling force, and in many cases it has yielded good fruit. The workers in all classes have been cheered by higher wages and greater means. The wiser amongst them are learning the advantages of saving for the first time. They may, and we hope will, accumulate sums which will place them and their families in a strong position when the war is ended and done with. Larger means have brought out with telling force the stimulating generosity which humbler folk invariably display in this country towards those less fortunate than themselves. There can be no doubt that both the worker and the giver of the latter type have since the war put on strength, and despite the zealous continuance of arduous labour they have for the most part attained to a higher standard of health and vigour than they have known before.

### Triumph of the Voluntary Principlle.

One of the most interesting facts in regard to money at the present time is the astonishment of the philanthropists at the war's revelation of the wealth of the nation and of many individuals. Those who are accustomed to cultivate the field of charity in all its ramifications, and to make themselves familiar with revenue and cost, are justified in their contention, made long before the war, that the voluntary principle would always produce adequate funds for every required and worthy enterprise, provided the appeal was addressed widely enough. The secret of the revelation in regard to money just referred to is that as the nation has been stirred up to realise that the very existence of the Empire, and of the country and nation, were at stake in the war, everybody has become interested in it, and all who were worthy of the name of patriot have been moved to offer themselves, as well as their means, to enable the enemy to be defeated and the nation and Empire saved. One good effect of the war has been the removal of pressure on the out-patient departments of the hospitals, and on the consulting-rooms of the physicians and surgeons throughout the country. The anxiety and excitement of war has brought the *malade imaginaire* face to face with realities with a round turn. He or she has given up taking physic, and substituted for it the health-giving practice of undertaking work within his or her compass, getting interested in it, and so losing all desire to pose as a human being afflicted with illness of a somewhat obscure but highly interesting character.

### A National Awakening.

The women of the nation in all classes have been splendid. They have found themselves, and their whole-hearted, self-denying, continuous devotion to the duty of co-operating with the men in every possible way everywhere with a view to expediting the successful termination of the war, is one of the most remarkable and pleasing facts that has ever happened in history. Both men and women, wealthy and otherwise, have in these last two years shed many false ideals. They, like our warriors, or many of them, have been awakened to the realisation of their spirituality, and the changes for good which all this movement and individual activity have brought to masses of people who formerly were imperilled by mere existence, is full of promise for the Empire and the nation. The death-rate has gone down, the health of the people of all classes has steadily improved, and the uniting of the nation into one huge family, where every member is interested in and desirous to help every other, must mean a new England after the war, and an Empire stronger and better and greater, more powerful, and, as we hope, more spiritual, than the earth has ever possessed in the past. Seeing that the characteristics of the British people that we have brought out are in fact identical with those to be met with in ever-increasing volume amongst the populations of the Empires and countries of our Allies, is it too much to hope that the Allied Nations will benefit to the same extent, and that the fruits of these great changes amongst the Allies may gradually spread to all nations to the uplifting of humanity and Christ's teaching throughout the whole world?

**Next week's article will deal with "The Pleasures of Life."**

